# Intrinsic resistance to tyrosine kinase inhibitors is associated with poor clinical outcome in metastatic renal cell carcinoma

**DOI:** 10.1186/1471-2407-11-295

**Published:** 2011-07-14

**Authors:** Jonas Busch, Christoph Seidel, Steffen Weikert, Ingmar Wolff, Carsten Kempkensteffen, Lisa Weinkauf, Stefan Hinz, Ahmed Magheli, Kurt Miller, Viktor Grünwald

**Affiliations:** 1 Department of Urology, Charité University Medicine Berlin, Charitéplatz 1, 10117 Berlin, Germany; 2 Department of Hematology, Hemostases, Oncology and Stem Cell Transplantation, Hannover Medical School, Carl-Neuberg-Strasse 1, 30625 Hannover, Germany

## Abstract

**Background:**

Data on sequential therapy in patients with metastatic renal cell carcinoma (mRCC) and intrinsic resistance to receptor tyrosine kinase inhibitor (rTKI) treatment remains vague.

**Methods:**

We retrospectively studied treatment characteristics and outcome of mRCC patients refractory to first rTKI therapy.

**Results:**

Thirty-five mRCC patients (male, 18; female, 11) with primary resistance to first rTKI therapy (sunitinib, n = 28; sorafenib, n = 7) and a median treatment interval of 2.4 months (1 - 4.6) were identified. In 22 patients, progressive disease (PD) was determined by a new metastatic lesion. Of these, 16 patients received subsequent therapy with 12 patients remaining refractory and 4 patients achieving disease stabilization. In 13 patients continuous growth of existing metastatic lesions determined PD. Of these, 9 received sequential therapy with 6 achieving disease stabilization. Altogether, 25 patients were treated sequentially (rTKI: n = 15; mTOR-inhibitor: n = 10) and achieved a median PFS of 3.2 months (range, 1-16.6). Fifteen patients failed to respond to either line of therapy. Disease control was not associated with type of subsequent therapy. Median OS was 14.9 months (CI: 5.5-24.4).

**Conclusion:**

Intrinsic resistance to rTKI is associated with a low chance of response to sequential therapy and a poor prognosis in mRCC patients.

## Background

During the last years potent therapeutic options evolved for patients with mRCC [1-4]. The introduction of targeted agents has significantly improved the treatment perspectives and prognosis of these patients. The majority of patients with good and intermediate prognosis according to the MSKCC criteria are treated with rTKI, particularly sunitinib, based on the results of two meta-analyses [5,6]. In spite of this progress in treatment options, a relevant subset of patients remains refractory to first rTKI therapy. On a molecular level, rTKIs target the vascular endothelial growth factor (VEGF) pathway to induce hypoxia, thereby inhibiting tumor growth. However, recent reports suggest that hypoxia may also select for a more malignant RCC phenotype, which may aggregate metastatic development and prone cells to insensitivity for antiangiogenic treatment [7]. Another possible explanation for the resistance to rTKI treatment could be attributed the fact that tumor cells can overcome the noxious rTKI hypoxic microenvironment by switching to invasive epithelial-mesenchymal transition [8].

The phenomenon of intrinsic resistance to rTKI is not well understood, and it remains unclear whether patients primary refractory to rTKI might benefit from other treatment regimens during sequential therapy. In the present study we aimed to characterize patients with intrinsic resistance to rTKI treatment and analyse their susceptibility to sequential therapy.

## Methods

We retrospectively reviewed the records of 189 patients treated with first line rTKI therapy (sunitinib or sorafenib) for mRCC at two large German academic centers. Medical records were retrieved and analyzed retrospectively in accordance with the regulatory agreement of the local ethics committee and the Declaration of Helsinki, approved by the local ethics committee. Thirty-five patients (18.5%) who had progressive disease (PD) as best response were considered intrinsic resistant and eligible for further analyses. Patient characteristics are shown in detail in table 1. All patients clearly experienced a progressive disease without any sign of mixed response regardless whether a new metastatic lesion developed or not. Second line targeted therapy (SL) consisted of another TKI (sunitinib or sorafenib) or an mTOR inhibitor (everolimus, temsirolimus). Third and fourth line therapy was individualized and included dovitinib, alpha-interferon, or bevacizumab plus alpha-interferon. Any therapy prior to first rTKI treatment was not counted as first line therapy but as previous therapy. Eight (22.9%) patients were treated on first rTKI therapy within prospective trials, and 17 (48.6%) patients were at least once treated within a prospective trial.

**Table 1:** Patients' characteristics

		n (%)
Sex	male	26 (74.3)
	female	9 (25.7)
	total	35 (100)
MSKCC risk	favourable	2 (5.7)
	intermediate	21 (60.0)
	poor	4 (11.4)
	unknown	8 (22.9)
ECOG status	0	21 (60)
	1	9 (25.7)
	> 1	1 (2.9)
	unknown	4 (11.4)
Histology	clear cell	29 (82.7)
	papillary	4 (11.4)
	other	2 (5.9)
Previous immunotherapy		14 (40)

Abbreviations:

MSKCC - Memorial Sloan-Kettering Cancer Center;

ECOG - Eastern Cooperative Oncology Group;

rTKI - receptor tyrosine kinase inhibitor.

Sunitinib was administered daily either as 50 mg or 37.5 mg orally over 4 weeks followed by a two week wash-out period. Sorafenib was administered continuously at a full dose of 400 mg orally twice a day. Dosing for everolimus was 10 mg daily and for temsirolimus 25 mg intravenously once weekly. All agents were administered until disease progression, death, or intolerable toxicity. Objective response was determined every second cycle of sunitinib or every two to three months for all other agents according to the standard Response Evaluation Criteria in Solid Tumors (RECIST) [9]. PFS and OS were calculated from initiation of first rTKI therapy using the Kaplan-Meier-method.

Furthermore, potential relationships between patients' characteristics (age, gender, MSKCC risk and ECOG performance group) with best response on SL and number of affected organ sites, new metastatic site at PD, localisation of PD and time to progression on prior treatment were assessed in an exploratory manner [10].

### Statistics

Associations between main characteristics and response to treatment were explored using the chi-square test/Fisher's exact test. A p-value < 0.05 was considered statistically significant. PFS and OS were estimated using the Kaplan-Meier method with the log-rank test.

## Results

**Figure 1:**
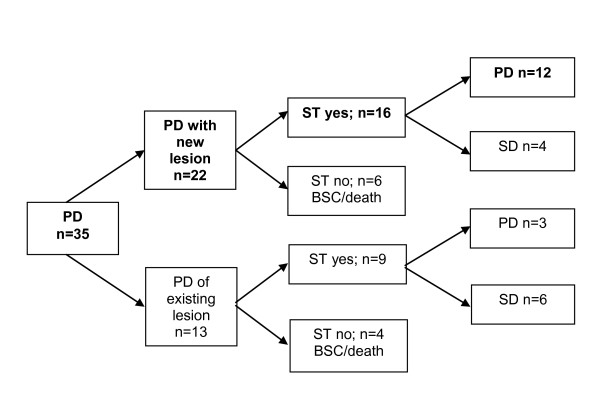
**Disease course of mRCC patients refractory to first rTKI treatment**. PD - progressive disease; SD - stable disease; ST - sequential therapy; BSC - best supportive care; rTKI - receptor tyrosine kinase inhibitor.

Thirty-five mRCC patients (18 male and 11 female) primary resistant to rTKI therapy with a median age of 62 years (range 39 - 79 years) were identified. All patients had radical nephrectomy prior to systemic therapy. Fourteen patients received immunotherapy before treatment with targeted agents over a median period of 3.6 months (range 0.5-10.3). Best response upon immunotherapy was stable disease in six patients. Initial rTKI therapy included sunitinib (n = 28) and sorafenib (n = 7). The median overall duration of first rTKI treatment was 2.4 (1-4.6; 95%CI: 2.2-2.6) months, specifically 2.5 (1-4.6; 95% CI: 2.3-2.7) months for sunitinib and 1.7 (1-3.2; 95% CI: 1.1-2.3) months for sorafenib (p = 0.130). The median PFS of the 25 patients who underwent sequential treatment with a targeted agent was 3.2 months (1 -16.6; 95% CI: 1.7-4.6). The remaining 10 patients did not receive any additional targeted therapy. Eight of them died of the disease after a median OS of 3.0 (95% CI: 1-5.1) months and 2 patients remained on best supportive care (BSC). Table 2 depicts the objective response assessment according to the line of targeted therapy. MSKCC prognosis group, the location or the number of metastases as well as the type of targeted agent were not associated with response to sequential therapy on fisher's exact test (data not shown). In 22 patients, PD during first rTKI therapy was determined by the occurrence of a new metastatic lesion. Of these, 16 patients received sequential treatment. 12 patients remained refractory to subsequent therapy while four patients had disease stabilization (mTOR: n = 2; rTKI: n = 2). 13 patients had PD by continuous growth of the initial metastatic lesions. Of these, 9 patients received sequential treatment, and 6 patients achieved disease stabilization (mTOR: n = 3; rTKI: n = 3) (Figure 1). Disease control (PR or SD) by any of the sequential therapy lines was achieved in 15 patients (42.8%). Only 14 and 7 patients received a third and fourth line of targeted treatment with a median estimated PFS of 1.9 (1-15; 95% CI: 1-3) and 2.6 (1-9; 95% CI: 2.1-3.1) months, respectively. Overall, only one patient accomplished a partial response on sequential treatment with temsirolimus. The median OS from the first rTKI therapy was 14.9 (95% CI: 5.5-24.4) months. Patients with new metastatic lesions on first rTKI therapy had a shorter, however statistically not significant, median OS of 10.7 (95% CI: 10.9-27.2) months than patients with growth of the initial lesions achieving a median OS of 19.1 (95% CI: 1.8-19.6) months (p = 0.161).

**Table 2:** Response assessment according to line and type of targeted therapy

Line of targeted therapy	PFS in months (range)		All	Sun	Sor	Eve	Tem	Other
1^st^	2.4	PR						
	(1-4.6)	SD						
		PD	35	28	7			
2^nd^	3.2	PR						
	(1-16.6)	SD	10	1	4	3	2	
		PD	15	2	8	4	1	
3^rd^	1.9	PR	1				1	
	(1-15)	SD	4		1	1		2
		PD	9			6	1	2
4^th^	2.6	PR						
	(1-9)	SD	4			1		3
		PD	3		1		1	1

**Abbreviations:**PFS - progression free survival; Sun - sunitinib; Sor - sorafenib; Eve - everolimus; Tem - temsirolimus; PR - partial response; SD - stable disease; PD - progressive disease

## Discussion

In this study we report on 35 patients with intrinsic resistant mRCC to rTKI treatment. This subset of patients seems to be characterized by a low likelihood of response to any form of available targeted therapy, with mTOR inhibitors being equally inefficacious as switching to another TKI. The median OS from initiation of first rTKI therapy was only 14.9 months and the median PFS upon second line targeted therapy was limited to 3.5 months with a disease control rate of roughly 40% in the sequence setting. The primary development of a new metastatic lesion during first rTKI treatment may indicate a particularly unfavourable prognosis.

In spite of recent substantial advances in treatment options the salvage strategy for patients with intrinsic resistance to rTKI treatment is not well defined. According to our data this subgroup of patients may have a low chance of overcoming rTKI resistance or responding to the available sequential treatment options. The rationale for sequential therapy with mTOR inhibitors in rTKI refractory patients lies in the expectation that resistance of the tumor to rTKI treatment may be reversed by targeting a different signaling pathway [11,12]. In the placebo-controlled phase 3 RECORD-1 trial everolimus turned out to be an efficacious treatment option following failure of rTKI therapy [2]. However, disease control can be achieved by rTKI treatment in the majority of treatment-naïve mRCC patients, suggesting underlying sensitivity to VEGFR-targeted agents. Whether mTOR inhibitors exert superior clinical activity in intrinsic resistant disease remains unknown.

Nonetheless, prospective data on the best sequential therapy among the available sequence options in mRCC are still lacking. In their retrospective study Vickers et al. reported that patients receiving another VEGF targeted therapy in the sequence setting may experience a longer progression free survival compared to those treated with mTOR inhibitors [13]. However, among the 216 analyzed patients with a second line therapy only 24 were treated with mTOR inhibitors and as few as three of them received everolimus, so that no valid comparison can be made between everolimus or other options in this setting. In another study Garcia et al. observed a PFS of 4.4 months in 49 patients treated with sorafenib following progression on either sunitinib or bevacizumab [14]. However, the vast majority of these patients had some benefit from first line therapy and developed resistance after several treatment cycles. Compared to these studies our data is based exclusively on patients with intrinsic rTKI resistance. In this subset of patients we observed no convincing efficacy of either of the common sequence therapy regimens. The small number of patients and the retrospective nature of our analysis limit the validity of our observations. The data from prospective randomized trials will certainly clarify some of these issues. However, our data indicate that there is a substantial subset of patients who will not respond to either targeted therapy option available today.

In general, sensitivity to targeted agents occurs when the tumor depends on the constitutive activity of signaling pathways for growth and progression. On the other hand resistance may develop when genetic alterations make the targeted proteins inaccessible to drug binding, activate alternative signaling pathways or upregulate molecule expression to compensate for the inhibition. Indeed, two general modes of resistance to angiogenesis inhibitors targeting the VEGF pathway have been proposed: adaptive (evasive) resistance, which occurs after a period of tumor control, and intrinsic (pre-existing) non-responsiveness without any therapeutic benefit [15]. Alternative pro-angiogenic signaling pathways within the tumor, recruitment of bone marrow-derived pro-angiogenic cells, increased protection of tumor vasculature by pericytes, and increased tumor cell invasiveness to escape oxygen and nutrient deprivation may all constitute escape mechanisms in response to therapy or in response to the selective pressures of the tumor microenvironment during malignant progression [15]. Each targeted agent including the various VEGF pathway inhibitors can cause a different compensatory tumor response, explaining at least in some parts the lack of cross-resistance and the potential benefit of re-challenge strategies [16].

Despite the proposed common deficiency of VHL function in clear cell RCC, distinct clinical outcome has been reported with current targeted therapies. These findings suggest that underlying genetic abnormalities may be more complex than previously assumed. A recent article by Gordan addressed this crucial question and suggested that HIF2 enhances c-MYC activity and promotes tumor progression in VHL deficient tumors [17].

In tumors with acquired resistance, distinct mechanisms of resistance have been detected [8,18,19]. Furthermore, additional genetic abnormalities have been reported in mRCC. The chromatin remodelling complex gene PBRM1 has been found to be mutated in 41% of 227 clearcell RCC cases [20]. The functional role of PBMR1 in mRCC remains to be determined, but these findings support the notion of a genetic heterogeneity in clear cell mRCC, which may determine intrinsic resistance in mRCC.

However, in intrinsic non-responsiveness the activation of alternative pathways (e.g. TIE2/ANG-2) may be of key relevance [3,21]. The currently used mTOR inhibitors, i. e. rapalogs, selectively target mTOR complex (TORC)-1, but leave TORC-2 unaffected. Developing inhibitors that target the kinase activity in both TORC1 and TORC2 could result in increased antitumor effects and overcome some of the obstacles associated with the TORC-1 inhibitors. Moreover, mTOR/S6 K activation, insulin receptor substrates 1 and 2 (IRS-1 and IRS-2) and insulin growth factor-1 (IGF-1) signaling, which all result in increased IGFR/PI3K/Akt signaling, appear as attractive targets for further drug development [22].

Current treatment strategies remain unsatisfactory in patients with intrinsic resistance to VEGF targeted therapies and underscore the medical need to advance the treatment for these patients. Based on the preliminary evidence of different genetic abnormalities in mRCC, clinical trials with agents that interfere with HIF-signalling or the chromatin remodelling complex seem suitable for patients with intrinsic resistant mRCC. However, gemcitabine-based chemotherapy has also been reported effective in single patients and may represent a distinct approach to intrinsic resistance in RCC [23]. Certainly, this subgroup of patients should be explored as a separate entity in clinical trials. Remarkably, the overall patients' prognosis of our study in terms of PFS or OS seems to be comparable to patients of the primary poor prognosis group according to MSKCC criteria.

## Conclusion

In conclusion, primary or intrinsic resistance to rTKI therapy indicates a poor prognosis, particularly if new metastatic sites develop. rTKI refractory mRCC patients have a low chance to respond to sequential therapy irrespective of the type of treatment. Further characterization of deregulated key signalling pathways in refractory RCC appears of utmost importance for improving the treatment perspectives. Other potential countermeasures to overcome resistance include combination of VEGF or mTOR inhibition with other signalling inhibitors or with cytotoxic agents.

## Competing interests

The authors declare that they have no competing interests.

## Authors' contributions

JB and CS did the data analyses and interpretation. JB, CS, SW and VG were involved in the conception and design of the study. JB, CS, CK, SH, IW, LW, AM and KM were involved in the provision of clinical data. JB and SW were in charge of the statistical design of the study. JB, SW, VG and CS wrote the manuscript.

SW and VG approved the final version. All authors read and approved the final manuscript.

## Pre-publication history

The pre-publication history for this paper can be accessed here:

http://www.biomedcentral.com/1471-2407/11/295/prepub

## References

[B1] Escudier B Pluzanska A Koralewski P Ravaud A Bracarda S Bevacizumab plus interferon alfa-2a for treatment of metastatic renal cell carcinoma: a randomised, double-blind phase III trial Lancet 2007 370 9605 2103 11 10.1016/S0140-6736(07)61904-7 18156031

[B2] Motzer RJ Escudier B Oudard S Hutson TE Porta C Efficacy of everolimus in advanced renal cell carcinoma: a double-blind, randomised, placebo-controlled phase III trial Lancet 2008 372 9637 449 56 10.1016/S0140-6736(08)61039-9 18653228

[B3] Motzer RJ Hutson TE Tomczak P Michaelson MD Bukowski RM Sunitinib versus interferon alfa in metastatic renal-cell carcinoma N Engl J Med 2007 356 2 115 24 10.1056/NEJMoa065044 17215529

[B4] Sternberg CN Davis ID Mardiak J Szczylik C Lee E Pazopanib in locally advanced or metastatic renal cell carcinoma: results of a randomized phase III trial J Clin Oncol 2010 28 6 1061 8 10.1200/JCO.2009.23.9764 20100962

[B5] Mills EJ Rachlis B O'Regan C Thabane L Perri D Metastatic renal cell cancer treatments: an indirect comparison meta-analysis BMC Cancer 2009 9 34 10.1186/1471-2407-9-34 19173737 PMC2637892

[B6] Thompson Coon JS Liu Z Hoyle M Rogers G Green C Sunitinib and bevacizumab for first-line treatment of metastatic renal cell carcinoma: a systematic review and indirect comparison of clinical effectiveness Br J Cancer 2009 101 2 238 43 10.1038/sj.bjc.6605167 19568242 PMC2720220

[B7] Yu JL Rak JW Coomber BL Hicklin DJ Kerbel RS Effect of p53 status on tumor response to antiangiogenic therapy Science 2002 295 5559 1526 8 10.1126/science.1068327 11859195

[B8] Loges S Mazzone M Hohensinner P Carmeliet P Silencing or fueling metastasis with VEGF inhibitors: antiangiogenesis revisited Cancer Cell 2009 15 3 167 70 10.1016/j.ccr.2009.02.007 19249675

[B9] Eisenhauer EA Therasse P Bogaerts J Schwartz LH Sargent D New response evaluation criteria in solid tumours: revised RECIST guideline (version 1.1) Eur J Cancer 2009 45 2 228 47 10.1016/j.ejca.2008.10.026 19097774

[B10] Motzer RJ Mazumdar M Bacik J Berg W Amsterdam A Survival and prognostic stratification of 670 patients with advanced renal cell carcinoma J Clin Oncol 1999 17 8 2530 40 10561319 10.1200/JCO.1999.17.8.2530

[B11] Hutson TE Bukowski RM Cowey CL Figlin R Escudier B Sequential use of targeted agents in the treatment of renal cell carcinoma Crit Rev Oncol Hematol 2010 10.1016/j.critrevonc.2010.07.018 20705477

[B12] Lane HA Wood JM McSheehy PM Allegrini PR Boulay A mTOR inhibitor RAD001 (everolimus) has antiangiogenic/vascular properties distinct from a VEGFR tyrosine kinase inhibitor Clin Cancer Res 2009 15 5 1612 22 10.1158/1078-0432.CCR-08-2057 19223496

[B13] Vickers MM Choueiri TK Rogers M Percy A Finch D Clinical outcome in metastatic renal cell carcinoma patients after failure of initial vascular endothelial growth factor-targeted therapy Urology 2010 76 2 430 4 10.1016/j.urology.2009.12.031 20223508

[B14] Garcia JA Hutson TE Elson P Cowey CL Gilligan T Sorafenib in patients with metastatic renal cell carcinoma refractory to either sunitinib or bevacizumab Cancer 2010 116 23 5383 90 10.1002/cncr.25327 20806321

[B15] Bergers G Hanahan D Modes of resistance to anti-angiogenic therapy Nat Rev Cancer 2008 8 8 592 603 10.1038/nrc2442 18650835 PMC2874834

[B16] Zama IN Hutson TE Elson P Cleary JM Choueiri TK Sunitinib rechallenge in metastatic renal cell carcinoma patients Cancer 2010 116 23 5400 6 10.1002/cncr.25583 21105118

[B17] Gordan JD Lal P Dondeti VR Letrero R Parekh KN HIF-alpha effects on c-Myc distinguish two subtypes of sporadic VHL-deficient clear cell renal carcinoma Cancer Cell 2008 14 6 435 46 10.1016/j.ccr.2008.10.016 19061835 PMC2621440

[B18] Ebos JM Lee CR Kerbel RS Tumor and host-mediated pathways of resistance and disease progression in response to antiangiogenic therapy Clin Cancer Res 2009 15 16 5020 5 10.1158/1078-0432.CCR-09-0095 19671869 PMC2743513

[B19] Huang D Ding Y Zhou M Rini BI Petillo D Interleukin-8 mediates resistance to antiangiogenic agent sunitinib in renal cell carcinoma Cancer Res 2010 70 3 1063 71 10.1158/0008-5472.CAN-09-3965 20103651 PMC3719378

[B20] Varela I Tarpey P Raine K Huang D Ong CK Exome sequencing identifies frequent mutation of the SWI/SNF complex gene PBRM1 in renal carcinoma Nature 2011 469 7331 539 42 10.1038/nature09639 21248752 PMC3030920

[B21] Rini BI Atkins MB Resistance to targeted therapy in renal-cell carcinoma Lancet Oncol 2009 10 10 992 1000 10.1016/S1470-2045(09)70240-2 19796751

[B22] Hudes GR Targeting mTOR in renal cell carcinoma Cancer 2009 115 10 Suppl 2313 20 19402072 10.1002/cncr.24239

[B23] Richey SL Ng C Lim ZD Jonasch E Tannir NM Durable remission of metastatic renal cell carcinoma with gemcitabine and capecitabine after failure of targeted therapy J Clin Oncol 2011 29 8 e203 5 10.1200/JCO.2010.31.6091 21172884 PMC4468428

